# Sesquiterpenes from *Ambrosia artemisiifolia* and their allelopathy

**DOI:** 10.3389/fpls.2022.996498

**Published:** 2022-09-02

**Authors:** Zhixiang Liu, Nan Zhang, Xiaoqing Ma, Tong Zhang, Xuan Li, Ge Tian, Yulong Feng, Tong An

**Affiliations:** ^1^College of Plant Protection, Shenyang Agricultural University, Shenyang, China; ^2^College of Biological Science and Technology, Shenyang Agricultural University, Shenyang, China

**Keywords:** allelopathy, *Ambrosia artemisiifolia*, invasive plant, isolate, sesquiterpenes

## Abstract

*Ambrosia artemisiifolia*, an invasive plant, has seriously harmed the agricultural production, native ecosystems and human health. Allelopathy is an important reason for the successful invasion of this alien plant. However, the chemical basis, action effects, action mechanism and release pathway of its allelopathy remain unclear. To address these problems, four sesquiterpenes (**1**–**4**), consisting of three new sesquiterpenes (**1**–**2**, **4**), were isolated from the whole plant of *A. artemisiifolia* using a variety of column chromatography techniques, and identified using HR-ESIMS, 1D-NMR, 2D-NMR, and ECD. All the compounds exhibited different levels of inhibitory effects on three native plants (*Setaria viridis*, *Digitaria sanguinalis*, *Chenopodium album*) and one model plant (*Arabidopsis thaliana*), especially compound **1**. In addition, the preliminary action mechanism of active compound 1 was revealed by FDA/PI staining assay. Furthermore, the allelopathic substances **1**–**3** were released into environment through the root secretion pathway by UPLC-MS/MS analyses.

## Introduction

In recent decades, with the acceleration of globalization and world trade, the spread of invasive plants has been increasing, causing a serious threat to agricultural production and the ecological environment (Bonnamour et al., 2021). It is urgent to reveal the invasive mechanism of alien plants, which is necessary for risk assessment of invasive plants and to develop effective prevention methods. In recent years, the novel weapons hypothesis (NWH) has become a hot topic of research for investigating the invasion mechanism of alien plants (Callaway and Ridenour, 2004). The hypothesis suggests that the successful invasion of alien plants is largely due to their release of allelopathic substances that are relatively novel to native plants (Bais, 2003). The allelopathic substances are released into the environment through appropriate pathways, such as root secretion and rainwater leaching, to negatively affect the growth of native plants, enabling alien plants to gain advantages and promote their expansion into dominant single populations (Thorpe et al., 2009). Therefore, fully understanding the allelopathic effects of invasive plants on native plants may help reveal the invasion mechanism of alien plants while also clarifying the chemical relationship between invasive plants and native plants, and this may enable new approaches for the utilization of invasive plants.

*Ambrosia artemisiifolia* L. (Asteraceae), which originated from North America, is an invasive weed found all over the world. The invader can inhibit the growth of native plants, destroying the original ecosystem and reducing agricultural production (Sun and Roderick, 2019). Furthermore, its pollen can cause a series of allergic reactions and directly affect human health (Zhao et al., 2017). Field observations have shown that *A. artemisiifolia* often forms a dominant single population with few other plants around it. The reason is not only that *A. artemisiifolia* has strong growth characteristics, but also its successful allelopathic effects on native plants. Previous studies reported that various solvent extracts of *A. artemisiifolia* had significant inhibitory effects on the growth and seed germination of *Lactuca sativa*, *Lycopersicon esculentum*, *Zea mays*, and other plants (Lehoczky et al., 2011; Vidotto et al., 2013; Bonea et al., 2018). These studies also confirmed that allelopathy is a major reason for the successful invasion of *A. artemisiifolia.* However, most studies only consider the effects of crude extract of *A. artemisiifolia* on native plants, and the chemical basis, action effects, action mechanism and release pathway of its allelopathy remain unclear. Like most higher plants, *A. artemisiifolia* contains a large number of secondary metabolites. Among them, sesquiterpenoids are the main chemical components, including the eudesmane-type, germacrane-type, bisabolane-type and guaiane-type, which have complex and variable structural frameworks (Taglialatela-Scafati et al., 2012; Ding et al., 2015; An et al., 2019). Studies have shown that sesquiterpenoids play an important role in plant allelopathy, which can significantly inhibit plant growth. Similarly, sesquiterpenoids may also be the potential allelopathic substances for *A. artemisiifolia* (Bennett and Wallsgrove, 1994; Chadwick et al., 2013).

In this study, to clarify the chemical basis of allelopathy, we isolated and identified the secondary metabolites from the whole plant of *A. artemisiifolia*. And the allelopathic effects of isolated compounds on three native plants (*Setaria viridis*, *Digitaria sanguinalis*, *Chenopodium album*) and one model plant (*Arabidopsis thaliana*) were also examined. In addition, the preliminary action mechanism of active compound **1** was revealed by FDA/PI staining assay. Furthermore, the release pathway of allelopathic substances was analyzed by UPLC-MS/MS analyses.

## Materials and methods

### General

Column chromatography was carried out using silica gel (Qingdao Marine, China), MCI (Mitsubishi, Japan), and Sephadex LH-20 (GE Healthcare, Sweden). RP-HPLC isolation was performed using a 1,260 system (Agilent, United States) coupled with a 250 mm × 10 mm, 5 μm XDB-C_18_ column (YMC, Japan). UV spectra were determined using a 241 spectrophotometer (Perkin Elmer, United States). HR-ESIMS spectra were measured using a 6,545 Q-TOF spectrometer (Agilent, United States). NMR spectra were recorded using an AV-600 instrument (Bruker, Germany). GC analysis was performed using a 7890A system (Agilent, United States). ECD spectra were obtained using a MOS-450 detector (Bio-Logic, France). Cell viability analyses were performed using an A1 laser confocal microscope (Nikon, Japan). UPLC-MS/MS analyses were performed using a 6,545 LC/Q-TOF system (Agilent, United States) coupled with a 50 mm × 2.1 mm, 1.9 μm EC-C_18_ column (Agilent, United States).

### Plant material

The whole plant of *A. artemisiifolia* was collected from Shenyang, Liaoning Province, China (123° 48′ E, 42° 05′ N) in August 2020 and identified by Professor Bo Qu (Shenyang Agricultural University). A voucher specimen (20200811) was kept in the herbarium of Shenyang Agricultural University.

### Extraction and isolation

The air-dried and powdered *A. artemisiifolia* (50 kg) were extracted with 80% ethanol at room temperature (200 L × 3, 7 days each time). The concentrated ethanol extract (1,200 g) was partitioned continuously with petroleum ether (5 L × 3) and ethyl acetate (5 L × 3). The ethyl acetate fraction (230 g) was then subjected to silica gel column chromatography eluted with a gradient of dichloromethane:methanol (99:1, 98:2, 95:5, 90:10, 80:20, 70:30, *v*/*v*) to yield five subfractions (Fr. A–E). Fr. D (18 g) was subjected to MCI column chromatography eluted with a gradient of methanol:H_2_O (10:90, 30:70, 50:50, 70:30, *v*/*v*) to yield five subfractions (Fr. D-1–5). Fr. D-2 (2.1 g) was subjected to Sephadex LH-20 column chromatography eluted with isocratic of acetone to yield eight subfractions (Fr. D-2-1–8). Fr. D-2-3 (130 mg) was isolated using RP-HPLC (210 nm, 5.0 ml/min) eluted with isocratic of methanol:H_2_O (20:80, *v*/*v*) to yield compounds **1** (10.2 mg, *t*_R_ = 32.4 min), **2** (3.5 mg, *t*_R_ = 17.5 min) and **3** (4.6 mg, *t*_R_ = 57.9 min), respectively. Similarly, Fr. D-2-5 (64 mg) was isolated using RP-HPLC (210 nm, 5.0 ml/min) eluted with isocratic of methanol:H_2_O (15:85, *v*/*v*) to yield compound **4** (6.1 mg, *t*_R_ = 78.1 min).

Compound **1**: light yellow powder; UV (methanol) *λ*_max_ (log*ε*): 219 (0.54) nm; ECD (methanol) *λ*_max_ (Δ*ε*) 209 (−60.57), 228 (−11.81), 246 (+8.90) nm; HR-ESIMS at *m/z* 303.1206 [M + Na]^+^ (calcd for C_15_H_20_O_5_Na, 303.1208); ^1^H and ^13^C NMR data, see Table 1.
Compound **2**: light yellow oil; UV (methanol) *λ*_max_ (log*ε*): 214.05 (0.20) nm; ECD (methanol) *λ*_max_ (Δ*ε*) 226 (−54.63) nm; HR-ESIMS at *m/z* 321.1311 [M + Na]^+^ (calcd for C_15_H_22_O_6_Na, 321.1314); ^1^H and ^13^C NMR data, see Table 1.
Compound **3**: light yellow oil; UV (methanol) *λ*_max_ (log*ε*): 213 (0.32) nm; ECD (methanol) *λ*_max_ (Δ*ε*) 226 (−5.92) nm; HR-ESIMS at *m/z* 363.1417 [M + Na]^+^ (calcd for C_17_H_24_O_7_Na, 363.1420); ^1^H and ^13^C NMR data, see Table 1.
Compound **4**: light yellow oil; UV (methanol) *λ*_max_ (log*ε*): 306 (0.11) nm; ECD (methanol) *λ*_max_ (Δ*ε*) 231 (+32.86), 310 (+8.16) nm; HR-ESIMS at *m/z* 263.1289 [M + H]^+^ (calcd for C_15_H_19_O_4_, 263.1283); ^1^H and ^13^C NMR data, see Table 1.

**Table 1:** ^1^H (600 MHz) and ^13^C NMR (150 MHz) spectroscopic data of compounds **1–4** in methanol-*d*_4_.

Position	1		2		3		4	
	*δ*_C_	*δ*_H_	*δ*_C_	*δ*_H_	*δ*_C_	*δ*_H_	*δ*_C_	*δ*_H_
1	76.2	3.42 (1H, dd, 11.8, 4.4)	76.3	3.36 (1H, dd, 12.4, 4.0)	75.6	3.42 (1H, dd, 12.2, 3.9)	131.5	
2	41.1	2.12 (1H, m) 1.55(1H, q, 11.8)	36.5	1.89 (1H, m) 1.68 (1H, q, 12.4)	34.0	1.94 (1H, m) 1.73 (1H, q, 12.2)	30.4	1.40 (1H, m) 1.28 (1H, m)
3	70.5	3.97 (1H, dd, 11.8. 5.1)	76.4	3.48 (1H, dd, 12.4, 4.5)	78.2	4.72 (1H, dd, 12.2, 4.6)	207.2	
4	148.0		76.3		74.9		138.2	
5	53.9	1.72 (1H, d, 11.0)	57.7	1.33 (1H, d, 11.5)	57.6	1.45 (1H, d, 12.0)	165.2	
6	79.8	5.20 (1H, d, 11.0)	81.1	5.30 (1H, d, 11.5)	80.6	5.31 (1H, d, 12.0)	78.8	5.41 (1H, d, 11.1)
7	169.3		169.8		169.5		56.4	2.14 (1H, m)
8	23.1	3.05 (1H, m) 2.50 (1H, td, 14.1, 5.7)	23.3	3.30 (1H, m) 2.49 (1H, td, 14.2, 5.6)	23.2	3.04 (1H, m) 2.50 (1H, td, 14.0, 5.5)	74.1	4.15 (1H, m)
9	37.8	2.23 (1H, m) 1.28 (1H, m)	41.2	2.17 (1H, m) 1.24 (1H, m)	41.0	2.18 (1H, m) 1.27 (1H, m)	42.6	2.88 (1H, dd, 15.2, 3.1) 2.35 (1H, dd, 15.2, 3.1)
10	42.6		42.3		42.2		133.3	
11	123.7		123.6		123.9		42.5	2.75 (1H, m)
12	175.6		174.6		174.5		180.0	
13	54.0	4.28 (2H, s)	54.0	4.28 (2H, s)	54.0	4.29 (2H, s)	14.9	1.32 (3H, d, 7.0)
14	10.9	0.90 (3H, s)	13.5	1.07 (3H, s)	13.5	1.10 (3H, s)	9.6	1.96 (3H, s)
15	106.7	5.37 (1H, s) 5.10 (1H, s)	17.4	1.35 (3H, s)	18.1	1.44 (3H, s)	25.5	1.94 (3H, s)
1′					172.2			
2′					21.0	2.06 (3H, s)		

### ECD calculations

The ECD calculations of compounds **1**–**4** were conducted using Gaussian 09 (Liu et al., 2021). Firstly, the conformational analyses were initially performed using the MMFF94 force field. Then, the obtained conformations were further optimized at the B3LYP/6-31G (d) level. Subsequently, the optimized conformations were calculated using a TDDFT method at the B3LYP/6–311 + G (2d, p) level (methanol). Finally, based on the Boltzmann weighting of each conformer, the calculated ECD curves were generated.

### Allelopathic assay

The allelopathic assay was performed as a previously described method with some modifications (Li et al., 2019). Firstly, the tested compounds were dissolved with DMSO and added to different volumes of 1/2 MS medium, respectively, to obtain the medium containing 100, 50, and 25 μM compounds, and the blank control contained the same volume of DMSO. Then, the 2 ml of medium was removed into the each well of 6-well plates, respectively. Subsequently, the seeds of *S. viridis*, *D. sanguinali*, *C. album, A. thaliana* were sterilized with 0.1% HgCl_2_ and washed with sterilized water at least three times, respectively. After the medium had cooled naturally to a solid state, 8 to 10 sterilized seeds were evenly placed in a row on the medium and cultured vertically in an illumination incubator (three replicates per compound). Finally, when the roots of the blank control grew to the bottom of 6-well plates, the lengths of roots were measured. Inhibitory rate (%) was calculated as (L_C_ − L_T_)/Lc × 100%, where L_C_ and L_T_ were the average lengths of blank control and compound-treated roots, respectively. Logran, a common herbicide, was used as a positive control.

### Statistical analyses

The data of allelopathic assay were expressed as means ± SD of three replicates. One-way ANOVA was used to compare data between groups when the data followed a normal distribution. Differences were considered to be statistically significant if *p* < 0.05.

### Cell viability analyses

The cell viability analyses were performed as a previously described method with some modifications (Pan et al., 2001). After the allelopathic assay, the root tips of *A. thaliana* (0.5 cm) were stained with a mixture of 12.5 μg/ml FDA (fluorescein diacetate) and 5 μg/ml PI (propidium iodide) for 10 min. Then, the roots were washed with distilled water and placed on slides. Finally, the roots were observed using a laser confocal microscope (excitation at 480 nm and emission at 520 nm). Red and green fluorescence represented dead and living cells, respectively.

### Collection of root secretion and rainwater leaching

The collection of root secretion and rainwater leaching was performed as a previously described method with some modifications (Wang et al., 2015). The rhizosphere soil of *A. artemisiifolia* (200 g) was randomly collected at 5–10 cm depths and carefully picked out the roots. The soil samples were crushed to pass through a sieve (30 mesh). The sieved soil was extracted ultrasonically with methanol (1 L) at room temperature for 30 min. The extraction were then filtered and concentrated *in vacuo* to obtain the root secretion samples. The aerial part of *A. artemisiifolia* (10 living bodies) was washed with distilled water (5 L) for 10 min. The rinses were then collected and concentrated *in vacuo* to obtain the rainwater leaching samples.

### UPLC-MS/MS analyses

The identification and quantification of compounds **1**–**4** in the root secretion and rainwater leaching samples of *A. artemisiifolia* were performed by UPLC-MS/MS. The solution of root secretion and rainwater leaching were filtered through a millipore filter (0.22 μm) before UPLC-MS/MS analyses (flow rate of 0.4 ml/min; injection volume of 1 μl; column temperature of 30°C), respectively. The mobile phase consisted of water containing 0.1% formic acid (A) and methanol (B) was programmed as follows: 0–5 min, B 10%; 5–20 min, B from 10% to 50%. Positive ionization was performed using the following settings: gas temperature, 280°C; drying gas flow, 8 L/ min; nebulizer, 35 psi; sheath gas temperature, 320°C; sheath gas flow, 12 L/ min; fragmentor, 145 V; skimmer, 65 V; oct 1 RF Vpp, 750 V. The identification of compounds in the root secretion and rainwater leaching was determined by comparing the retention times, MS, MS/MS spectra with those of isolated compounds **1**–**4**. The quantification of compounds in the root secretion and rainwater leaching was performed using the same method described above, with the isolated compounds as external standards. The standard curves were plotted using six concentrations (100, 10, 1, 0.1, 0.01, 0.001 μg/ml) and the corresponding ion peak area. The regression equations of compounds **1**–**3** were *y* = 22,758*x* − 8415.2 (R^2^ = 0.9996), *y* = 17,790*x* + 7201.4 (R^2^ = 0.9996) and *y* = 20,732*x* − 5553.5 (R^2^ = 0.9997), respectively.

## Results and discussion

Compound **1** was obtained as a light yellow powder with C_15_H_20_O_5_ (six degrees of unsaturation) according to HR-ESIMS data (*m/z* 303.1206 [M + Na]^+^, calcd for C_15_H_20_O_5_Na, 303.1208). Its 1D-NMR spectra (Table 1) showed one carbonyl group [*δ*_C_ 175.6 (C-12)], two double bond groups [*δ*_H_ 5.37 (1H, s, H-15), 5.10 (1H, s, H-15); *δ*_C_ 169.3 (C-7), 148.0 (C-4), 123.7 (C-11), 106.7 (C-15)], one primary alcohol group [*δ*_H_ 4.28 (2H, s, H-13); *δ*_C_ 54.0 (C-13)], two secondary alcohol groups [*δ*_H_ 3.97 (1H, dd, *J* = 11.8, 5.1 Hz, H-3), 3.42 (1H, dd, *J* = 11.8, 4.4 Hz, H-1); *δ*_C_ 76.2 (C-1), 70.5 (C-3)], and one methyl group [*δ*_H_ 0.90 (3H, s, H-14); *δ*_C_ 10.9 (C-14)], respectively.

**Figure 1:**
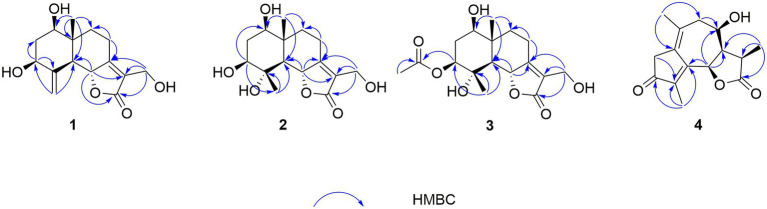
Key HMBC correlations of compounds **1–4**.

**Figure 2:**
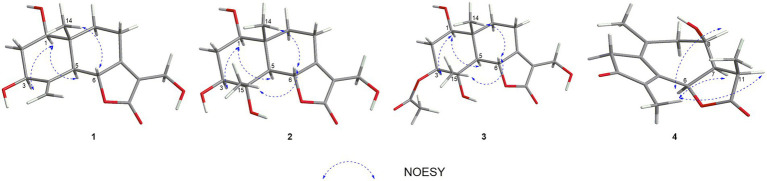
Key NOESY correlations of compounds **1–4**.

**Figure 3:**
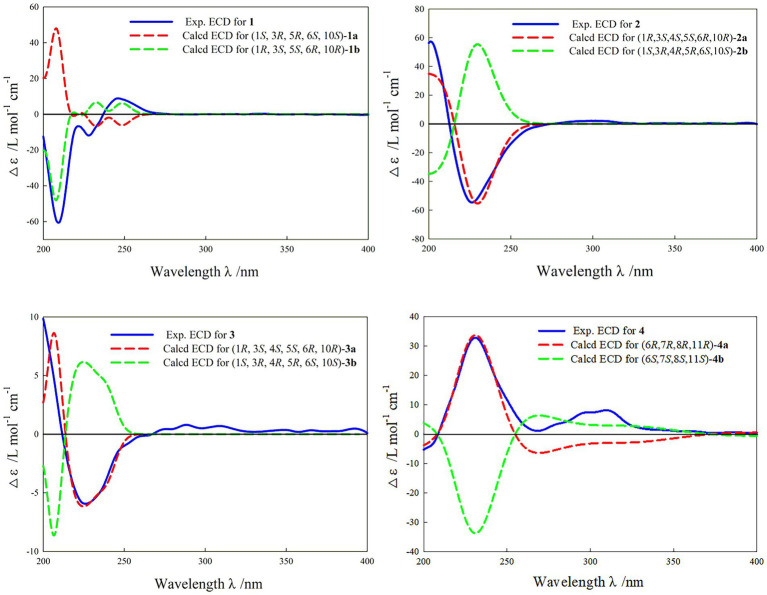
Experimental and calculated ECD curve of compounds **1–4**.

**Figure 4:**
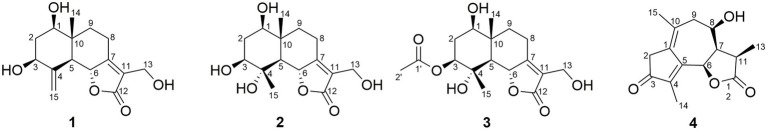
Structures of compounds **1–4**.

In the HMBC spectrum (Figure 1), the key correlations of H-3/C-2, C-4; H-6/C-5; H-8/C-7, C-9; H-14/C-1, C-5, C-9, C-10; H-15/C-3, C-5 established a eudesmane-type sesquiterpene moiety (An et al., 2019). The key correlations of H-6/C-11, C-12; H-13/C-7, C-11, C-12 established a isosiphonodin moiety (Caloprisco et al., 2002). In addition, the key correlations of H-6/C-5; H-8/C-7 revealed that the isosiphonodin moiety was connected to the eudesmane-type sesquiterpene moiety through C-6 and C-7. In the NOESY spectrum (Figure 2), the key correlations of H-1/H-3, H-5; H-14/H-6 indicated that H-1, H-3, H-5 and H-6, H-14 were oriented on the opposite side. Furthermore, the calculated ECD curve of (1*R*, 3*S*, 5*S*, 6*R*, 10*R*)-**1b** matched well with the experimental result of **1** (Figure 3). Thus, the planar and stereoscopic structure of compound **1** was constructed and named Eudesmanol A (Figure 4).

Compound **2** was obtained as a light yellow oil with C_15_H_22_O_6_ (five degrees of unsaturation) according to HR-ESIMS data (*m/z* 321.1311 [M + Na]^+^, calcd for C_15_H_22_O_6_Na, 321.1314). Comparison of its 1D NMR spectra with Eudesmanol A showed that they were very similar except for one additional methyl group [*δ*_H_ 1.35 (3H, s, H-15); *δ*_C_ 17.4 (C-15)], one additional tertiary alcohol group [*δ*_C_ 76.3 (C-4)], and one missing terminal double bond group. In the HMBC spectrum (Figure 1), the key correlations of H-15/C-4, C-5 revealed that the methyl and hydroxyl groups were connected to Eudesmanol A by C-4. In the NOESY spectrum (Figure 2), the key correlations of H-1/H-3, H-5; H-6/H-14, H-15 indicated that H-1, H-3, H-5 and H-6, H-14, H-15 were oriented on the opposite side. Furthermore, the calculated ECD curve of (1*R*, 3*S*, 4*S*, 5*S*, 6*R*, 10*R*)-**2a** matched well with the experimental result of **2** (Figure 3). Thus, the planar and stereoscopic structure of compound **2** was constructed and named Eudesmanol B (Figure 4).

Compound **3** was obtained as a light yellow oil with C_17_H_24_O_7_ (six degrees of unsaturation) according to HR-ESIMS data (*m/z* 363.1417 [M + Na]^+^, calcd for C_17_H_24_O_7_Na, 363.1420). Comparison of its 1D NMR spectra with a known compound 3*β-*Acetoxy-l*β*, 4*α*, 13-trihydroxyeudesm-7 (11)-*en*-6*α*, l2-olide showed that they were very similar except for the different deuterated solvents. In the HMBC spectrum (Figure 1), the key correlations revealed that compound **3** has the same planar structure as 3*β-*Acetoxy-l*β*, 4*α*, 13-trihydroxyeudesm-7 (11)-*en*-6*α*, l2-olide (Abdel-Mogi et al., 1989). In the NOESY spectrum (Figure 2), the key correlations of H-1/H-3, H-5; H-6/H-14, H-15 indicated that H-1, H-3, H-5 and H-6, H-14, H-15 were oriented on the opposite side. Furthermore, the calculated ECD curve of (1*R*, 3*S*, 4*S*, 5*S*, 6*R*, 10*R*)-**3a** matched well with the experimental result of **3** (Figure 3), which revealed that compound **3** has the same stereoscopic structure as 3*β-*Acetoxy-l*β*, 4*α*, 13-trihydroxyeudesm-7 (11)-*en*-6*α*, l2-olide. Thus, the planar and stereoscopic structure of compound **3** was constructed (Figure 4).

Compound **4** was obtained as a light yellow oil with C_15_H_18_O_4_ (seven degrees of unsaturation) according to HR-ESIMS data (*m/z* 263.1289 [M + H]^+^, calcd for C_15_H_19_O_4_, 263.1283). Its 1D-NMR spectra (Table 1) showed two carbonyl groups [*δ*_C_ 207.2 (C-3), 180.0 (C-12)], two double bond groups [*δ*_C_ 165.2 (C-5), 138.2 (C-4), 133.3 (C-10), 131.5 (C-1)], one secondary alcohol group [*δ*_H_ 4.15 (1H, m, H-8); *δ*_C_ 74.1 (C-8)], three methyl groups [*δ*_H_ 1.96 (3H, s, H-14), 1.94 (3H, s), 1.32 (3H, d, *J* = 7.0 Hz); *δ*_C_ 25.5 (C-15), 14.9 (C-13), 9.6 (C-14)], respectively.

In the HMBC spectrum (Figure 1), the key correlations of H-2/C-4; H-6/C-5, C-7, C-8; H-9/C-8; H-14/C-3, C-4, C-5; H-15/C-1, C-9 established a guaiane-type sesquiterpene moiety (Ding et al., 2015). The key correlations of H-6/C-7; H-13/C-7, C-11, C-12 established a *α*-methylbutyzolactone moiety (Simonaitis and Pitts, 1969). In addition, the key correlations of H-6/C-5, C-7, C-8 revealed that the *α*-methylbutyzolactone moiety was connected to the guaiane-type sesquiterpene moiety by C-6 and C-7. In the NOESY spectrum (Figure 2), the key correlations of H-7/H-6; H-8/H-6; H-11/H-6 indicated that H-6, H-7, H-8, H-11 and H-13 were oriented on the opposite side. Furthermore, the calculated ECD curve of (6*R*, 7*R*, 8*R*, 11*R*)-**4a** matched well with the experimental result of **4** (Figure 3). Thus, the planar and stereoscopic structure of compound **4** was constructed and named Guaianin (Figure 4).

**Figure 5:**
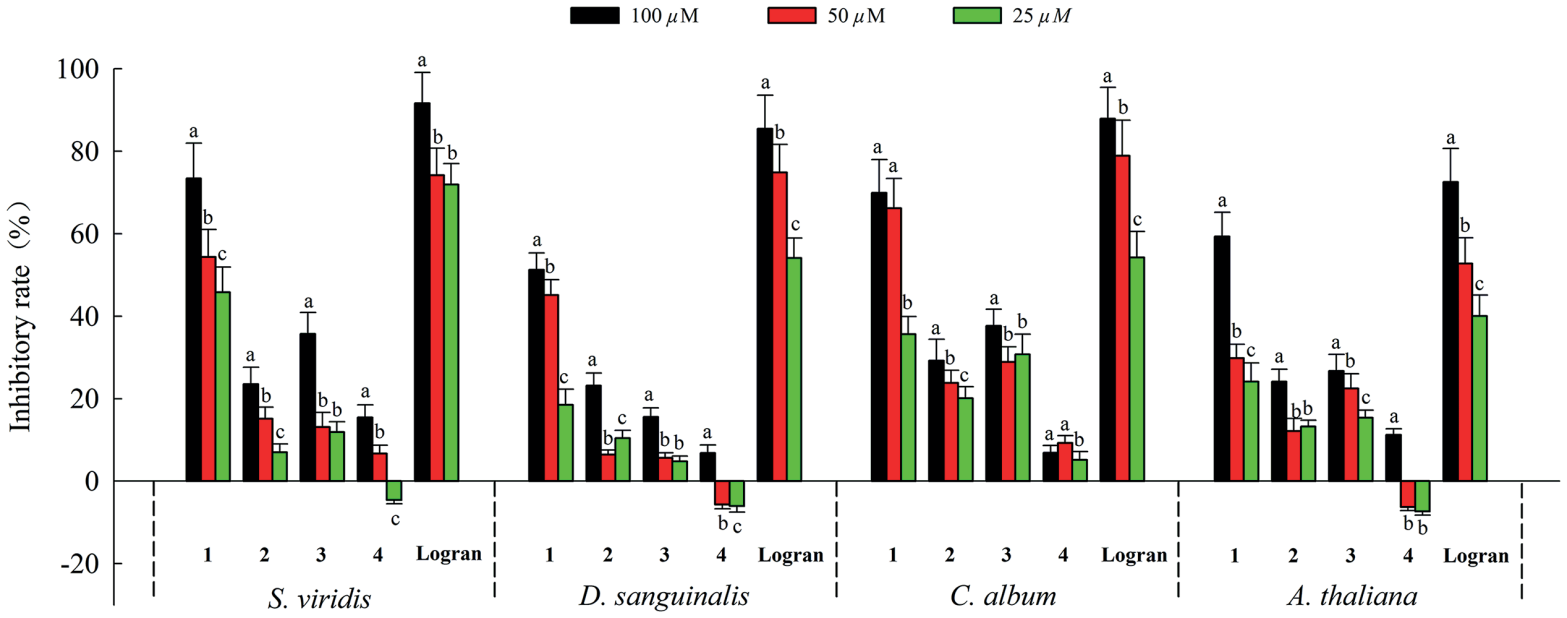
Allelopathic effects of compounds **1–4** on root growth of weeds. Different letters indicate significant differences between concentration treatments (*p* < 0.05).

Moreover, we assessed the allelopathic effects of compounds **1**–**4** from *A. artemisiifolia* on the root elongation of *S. viridis*, *D. sanguinali*, *C. album, A. thaliana.* As shown in Figure 5, all of the compounds exhibited different levels of allelopathic effects. Compounds **1**–**3** exhibited moderate allelopathic effects with inhibition rates ranging from 15.60% to 73.42% at 100 *μ*M. In addition, compound **4** exhibited slight allelopathic effects, and promoted the root elongation of three kinds of *S. viridis*, *D. sanguinali*, *A. thaliana* at low concentrations. Notably, compound **1** showed potent allelopathic effects (73.42% ± 8.54% on *S. viridis*, 51.23% ± 4.12% on *D. sanguinali*, 69.88% ± 8.09% on *C. album*, 59.34% ± 5.80% on *A. thaliana,* respectively) with more than 50% inhibitory rate at 100 μM, which approached the results observed for Logran. Comparisons of the structure–activity relationships of compounds **1**–**3** showed that the eudesmane-type sesquiterpenes with a terminal double bond group at C-4 may have greater allelopathic effects than the compounds with hydroxyl and methyl groups at C-4.

**Figure 6:**
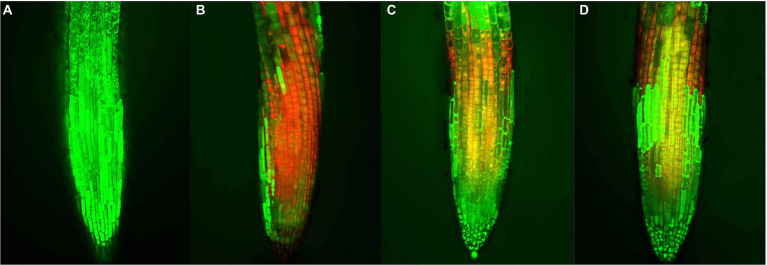
Effects of compound **1** on cell viability of the root tips of *Arabidopsis thaliana.* at concentrations of **(A)** 0, **(B)** 100, **(C)** 50, and **(D)** 25 μM, respectively. Red and green fluorescence represented dead and living cells, respectively.

When plants are subjected to external stress, the cell viability changes. Thus, cell viability can be used as an important indicator for evaluating the allelopathic effects of compounds on plants (Yan et al., 2016). The preliminary action mechanism of active compound **1** was revealed by FDA/PI staining assay. As shown in Figure 6, the root tips of *A. thaliana* began to show red fluorescence at 25 μM of compound **1**, indicating that compound **1** could cause the partial cell death of *A. thaliana* at a low concentration. Additionally, when the concentration of compound **1** was 100 μM, the red fluorescence was dominant and green fluorescence was reduced in the root tips of *A. thaliana*. The results showed that the cell viability of root tips of *A. thaliana* decreased with increasing concentrations of compound **1**. Therefore, we speculated that compound **1** could play an allelopathic role by decreasing the cell viability of plants. However, the reason why the plant cell viability was decreased by compound **1** needs to be explored further.

**Figure 7:**
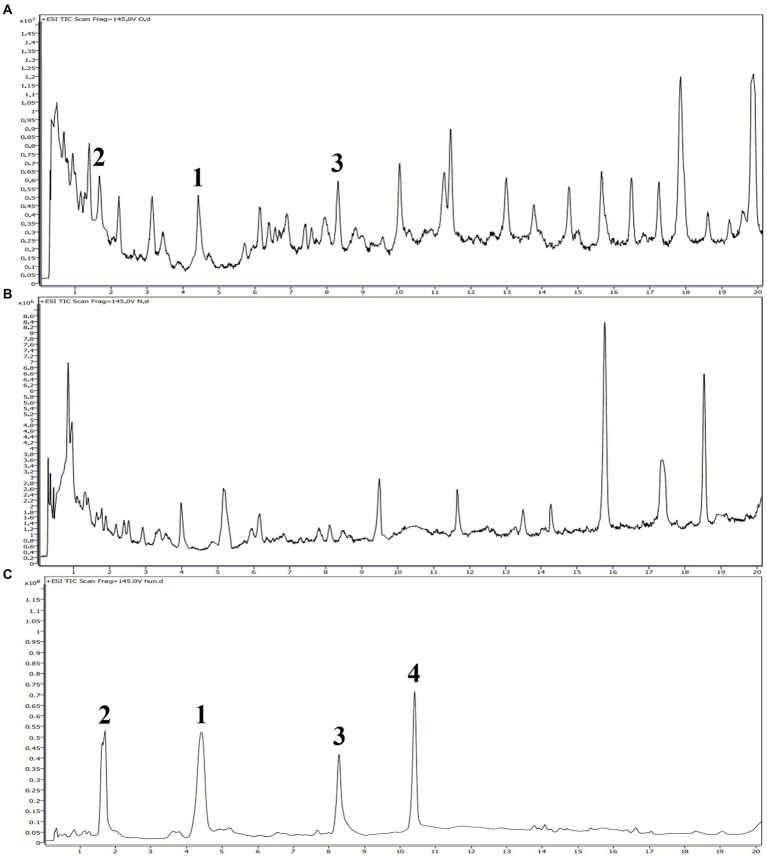
UPLC-MS/MS analyses of the root secretion, rainwater leaching, and isolated compounds from *Ambrosia artemisiifolia.* **(A)** TIC chromatogram of the root secretion from *A. artemisiifolia*; **(B)** TIC chromatogram of the rainwater leaching from *A. artemisiifolia*; **(C)** TIC chromatogram of compounds **1–4** from *A. artemisiifolia.*

It is well-known that allelopathic substances can play their allelopathic roles only when they are released into environment through appropriate pathways (Thorpe et al., 2009). To determine the allelopathic release pathway of the sesquiterpenes **1**–**4**, UPLC-MS/MS analyses were carried out to detect root secretion and rainwater leaching of *A. artemisiifolia*. According to their TIC chromatogram (Figure 7), compounds **1**–**3** had retention times of 4.42, 1.67, and 8.29 min, respectively, and they were detected in the root secretion of *A. artemisiifolia*., but no compounds were detected in rainwater leaching. The concentrations of compounds **1**–**3** in the rhizosphere soil were also determined as 0.55 ± 0.06, 0.44 ± 0.03, 0.48 ± 0.04 μg/g, respectively. The results indicated that the active compounds **1**–**3** were released into environment through root secretion to negatively affect the growth of native plants. Since the content of compounds **1**–**3** in the rhizosphere soil is low, we speculated that *A. artemisiifolia* could accumulate these compounds to achieve effective inhibitory concentrations only under certain conditions (e.g., when competing with other weeds; Kong et al., 2018). However, their dynamic release process remains unclear in the actual environment. Compound **4** was not detected in root secretion, possibly because of its rapid degradation by soil microorganisms or low water solubility. In addition, the lack of compounds in the rainwater leaching may be caused by the short washing times with distilled water, the replacement of distilled water for rainwater, or the absence of compounds **1**–**4** in the aerial part of *A. artemisiifolia*.

In recent years, invasive plants have brought serious ecological, economic and social problems around the world. To effectively control these alien plants, it is of great theoretical and practical significance to clarify their invasion mechanisms (Enders et al., 2020). Numerous studies have shown that allelopathy is a driving factor in the successful invasion of alien plants (Yuan et al., 2021). For example, alien plants can release allelopathic substances that are relatively novel to native plants, thus negatively affecting on the growth of native plants. Sesquiterpenoids are abundant in many invasive plants, which play an important role in plant allelopathy and can significantly inhibit plant growth (Bennett and Wallsgrove, 1994; Chadwick et al., 2013) In our study, three eudesmane-type sesquiterpenes (**1**–**3**) with potent allelopathic effects were isolated and identified from *A. artemisiifolia.* These active compounds can be released into environment through the root secretion pathway to affect the cell viability of surrounding plants, thus promoting the invasion of *A. artemisiifolia.* We speculated that native plants have not yet adapted to these compounds (**1**–**3**) because of their novel structures and the lack of coevolution between alien and native plants. However, further research is needed to determine whether these compounds are already present in the original areas of invasive plants and how they specifically affect local plants. In addition, sesquiterpenoids generally have strong phytotoxic activities against many weeds (Anese et al., 2015; Masi et al., 2020). Moreover, allelopathic substances have the advantages of safety, easy degradation and no resistance to weed control (Macías et al., 2019). Therefore, the allelopathic sesquiterpenes (**1**–**3**) from *A. artemisiifolia* can be used to develop novel botanical herbicides and reduce the use of synthetic herbicides.

## Conclusion

In summary, four sesquiterpenes (**1**–**4**), consisting of three eudesmane-type sesquiterpenes, one guaiane-type sesquiterpene, were isolated from the whole plant of *A. artemisiifolia* by a variety of column chromatography techniques. Their planar and stereoscopic structures were identified using HR-ESIMS, 1D-NMR, 2D-NMR, and ECD. All the compounds exhibited different levels of allelopathic effects on three native plants (*S. viridis*, *D. sanguinalis*, *C. album*) and one model plant (*A. thaliana*), but in particular, compound **1** significantly inhibited the root elongation of plants at 100 *μ*M. In addition, active compound **1** decreased cell viability by exerting allelopathic effects, as observed by FDA/PI staining assay. Furthermore, the eudesmane-type sesquiterpenes (**1**–**3**) were mainly released into environment through the root secretion pathway, which was revealed by UPLC-MS/MS analyses. Our findings not only helped to reveal the invasion mechanism of *A. artemisiifolia* from the perspective of allelopathy, but also supported a promising strategy for its exploitation in herbicides.

## Data availability statement

The original contributions presented in the study are included in the article/Supplementary material, further inquiries can be directed to the corresponding authors.

## Author contributions

ZL designed the research, performed the experiments, analyzed the data, and wrote the manuscript. NZ, XM, TZ, XL, and GT performed the experiments and analyzed the data. YF and TA reviewed and revised the draft. All authors contributed to the article and approved the submitted version.

## Funding

This research was supported by the National Natural Science Foundation of China (31700472).

## Conflict of interest

The authors declare that the research was conducted in the absence of any commercial or financial relationships that could be construed as a potential conflict of interest.

## Publisher’s note

All claims expressed in this article are solely those of the authors and do not necessarily represent those of their affiliated organizations, or those of the publisher, the editors and the reviewers. Any product that may be evaluated in this article, or claim that may be made by its manufacturer, is not guaranteed or endorsed by the publisher.
