# Supplementary material for: Sesquiterpenes from Ambrosia artemisiifolia and their allelopathy
Source: Front Plant Sci. 2022 Sep 2;13:996498. doi: 10.3389/fpls.2022.996498 (PMC9478656; doi:10.3389/fpls.2022.996498)

**Supporting Information**

**Sesquiterpenes from *Ambrosia artemisiifolia* and their allelopathy**

Zhixiang Liu ^1,2^, Nan Zhang ^2^, Xiaoqing Ma ^2^, Tong Zhang ^2^, Xuan Li ^2^, Ge Tian ^2^, Yulong Feng ^1,2,*^, Tong An ^1,2,*^

^1^ College of Plant Protection, Shenyang Agricultural University, Shenyang 110866, P.R. China

^2^ College of Biological Science and Technology, Shenyang Agricultural University, Shenyang 110866, P.R. China

*Corresponding authors. Phone: +86-24-88487163. Fax: +86-24-88492799.

E-mail addresses: [fyl@syau.edu.cn](mailto:fyl@syau.edu.cn) (Y.-L. Feng); m18602463916@163.com (T. An).

**Figure. S1.**  The UV spectrum of compound **1**

**Figure. S2.** The HR-ESIMS spectrum of compound **1**

**Figure. S3.** The ^1^H-NMR spectrum of compound **1**

**Figure. S4.** The ^13^C-NMR spectrum of compound **1**

**Figure. S5.** The HSQC spectrum of compound **1**

**Figure. S6.** The HMBC spectrum of compound **1**

**Figure. S7.** The NOESY spectrum of compound **1**

**Figure. S8.** The UV spectrum of compound **2**

**Figure. S9.** The HR-ESIMS spectrum of compound **2**

**Figure. S10.** The ^1^H-NMR spectrum of compound **2**

**Figure. S11.** The ^13^C-NMR spectrum of compound **2**

**Figure. S12.**  The HSQC spectrum of compound **2**

**Figure. S13.** The HMBC spectrum of compound **2**

**Figure. S14.** The NOESY spectrum of compound **2**

**Figure. S15.**  The UV spectrum of compound **3**

**Figure. S16.** The HR-ESIMS spectrum of compound **3**

**Figure. S17.** The ^1^H-NMR spectrum of compound **3**

**Figure. S18.** The ^13^C-NMR spectrum of compound **3**

**Figure. S19.**  The HSQC spectrum of compound **3**

**Figure. S20.** The HMBC spectrum of compound **3**

**Figure. S21.** The NOESY spectrum of compound **3**

**Figure. S22.**  The UV spectrum of compound **4**

**Figure. S23.** The HR-ESIMS spectrum of compound **4**

**Figure. S24.** The ^1^H-NMR spectrum of compound **4**

**Figure. S25.** The ^13^C-NMR spectrum of compound **4**

**Figure. S26.** The HSQC spectrum of compound **4**

**Figure. S27.** The HMBC spectrum of compound **4**

**Figure. S28.** The NOESY spectrum of compound **4**

**Figure. S29.** The MS spectra of compounds **1** in the TIC chromatogram.

**Figure. S30.** The MS spectra of compounds **2** in the TIC chromatogram.

**Figure. S31.** The MS spectra of compounds **3** in the TIC chromatogram.

**Figure. S32.** The MS spectra of compounds **4** in the TIC chromatogram.

**Figure. S33.** The MS/MS spectra of compounds **1** in the TIC chromatogram.

**Figure. S34.** The MS/MS spectra of compounds **2** in the TIC chromatogram.

**Figure. S35.** The MS/MS spectra of compounds **3** in the TIC chromatogram.

**Figure. S36.** The MS/MS spectra of compounds **4** in the TIC chromatogram.

**Figure. S1.**


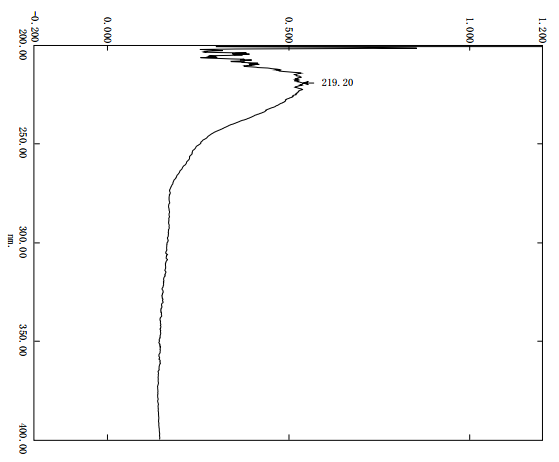


**Figure. S2.**


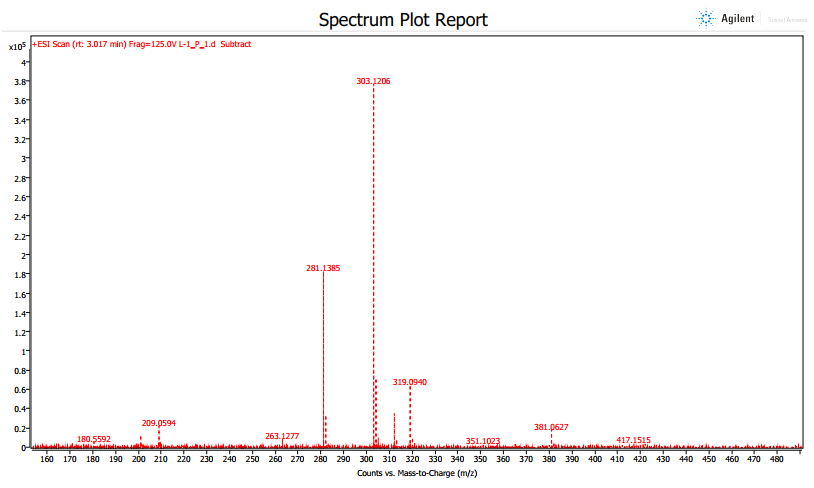


**Figure. S3.**


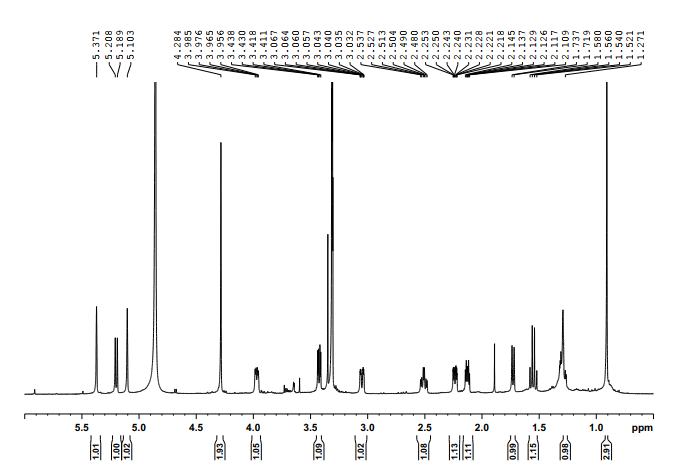


**Figure. S4.**


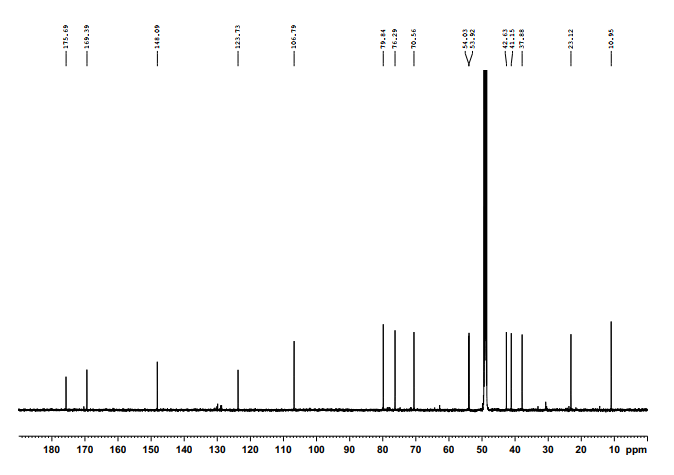


**Figure. S5.**


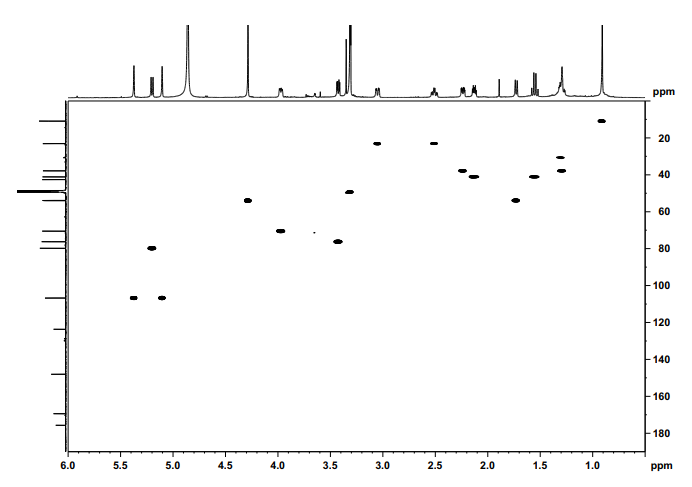


**Figure. S6.**


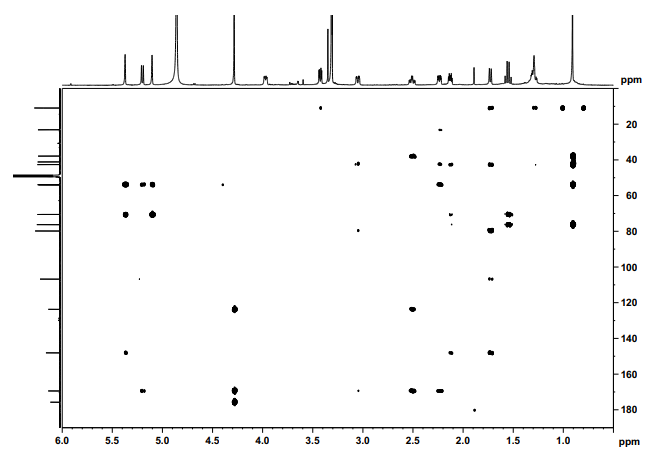


**Figure. S7.**


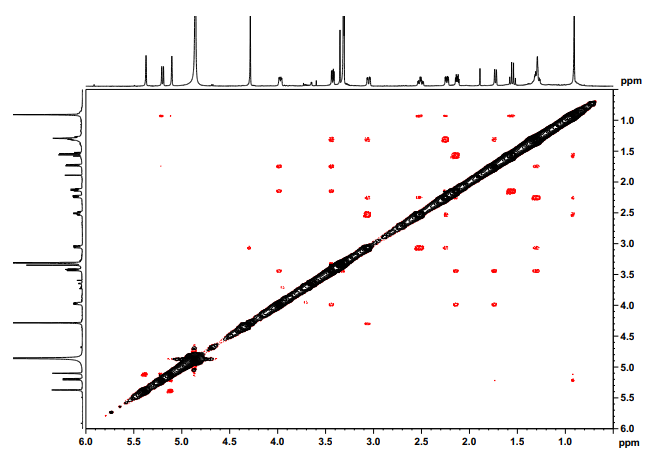


**Figure. S8.**


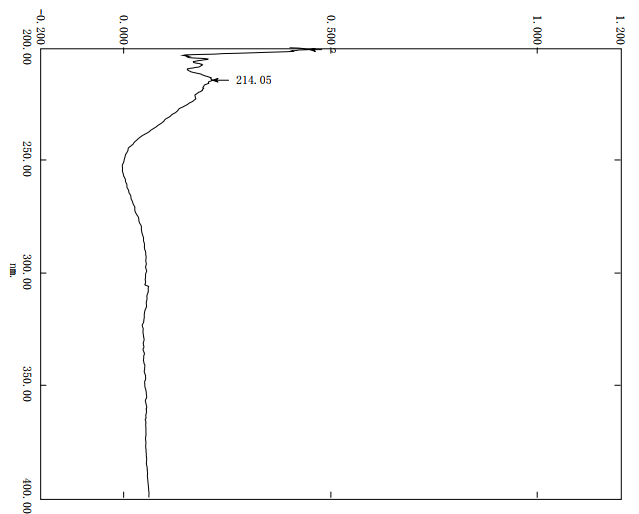


**Figure. S9.**


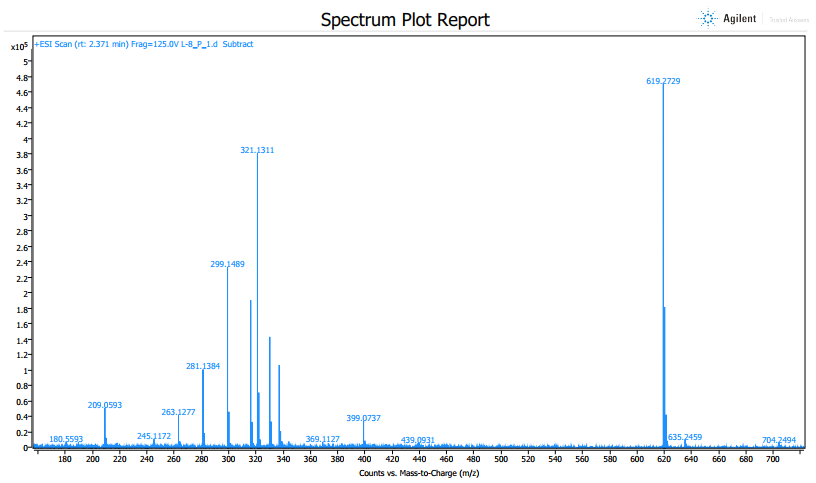


**Figure. S10.**


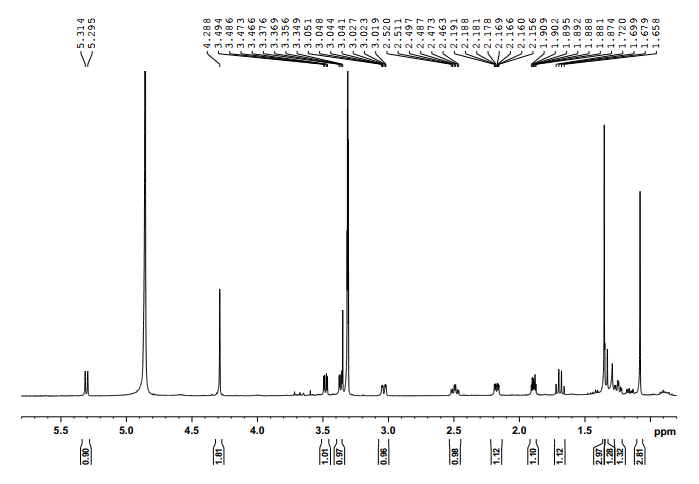


**Figure. S11.**


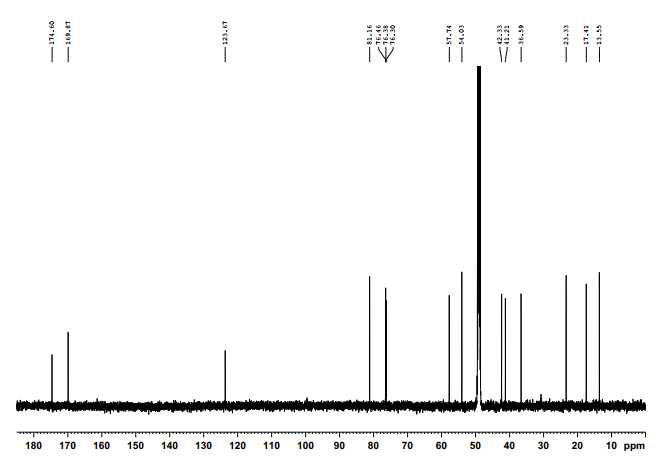


**Figure. S12.**


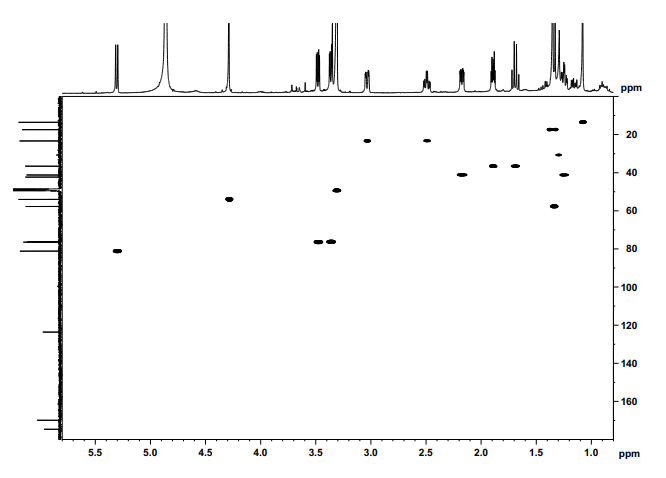


**Figure. S13.**


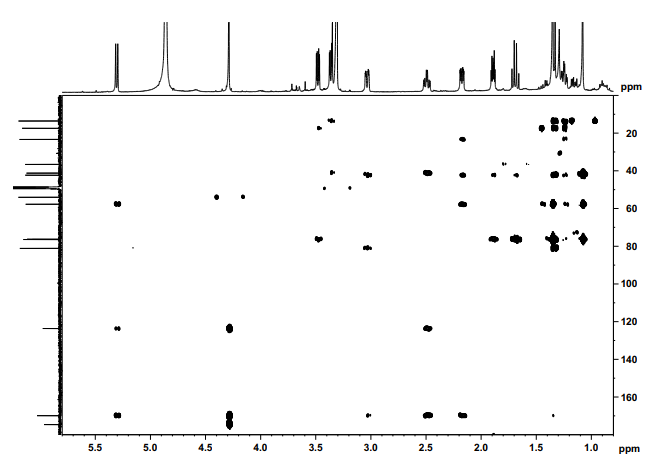


**Figure. S14.**


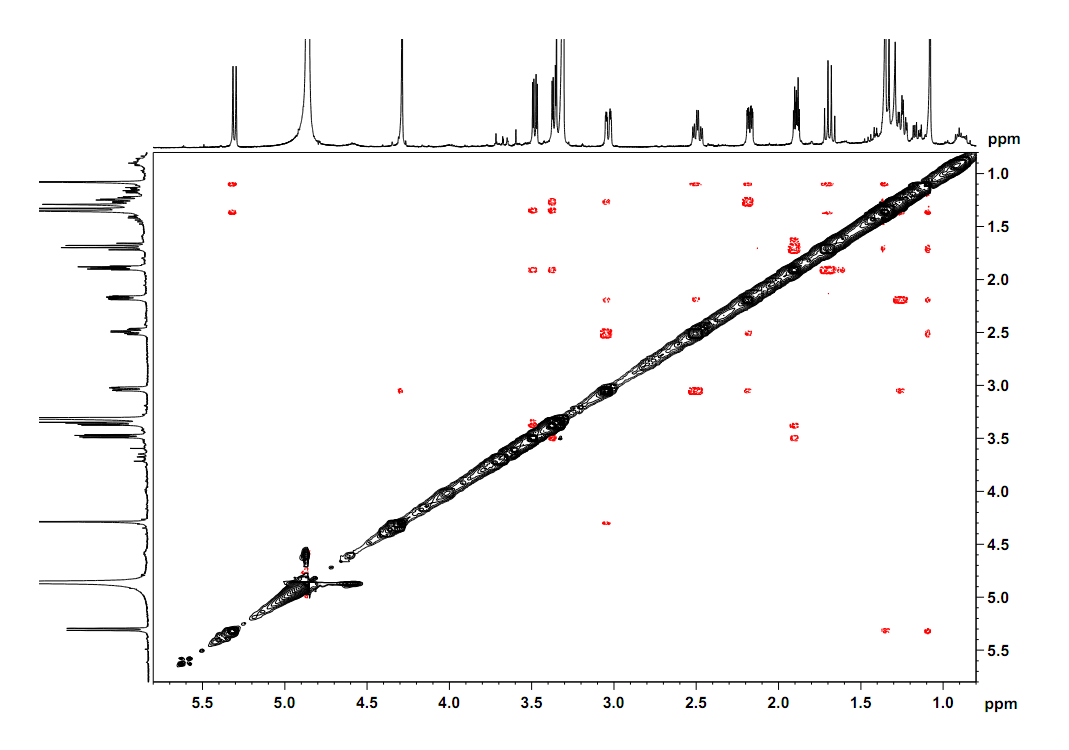


**Figure. S15.**


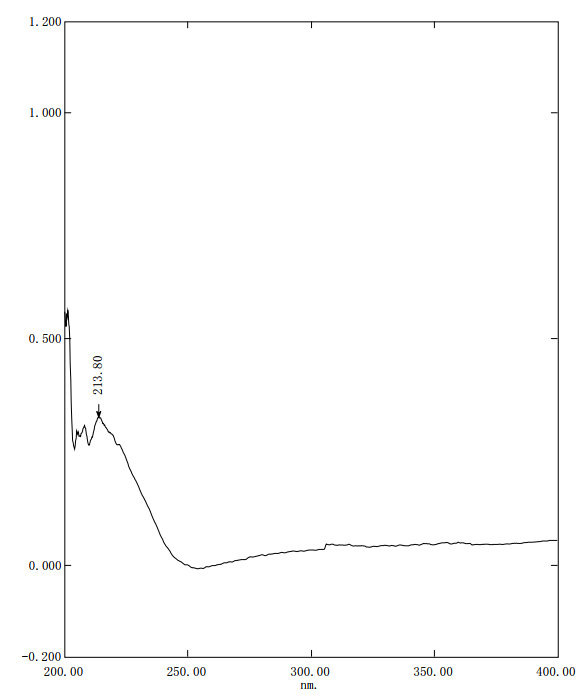


**Figure. S16.**


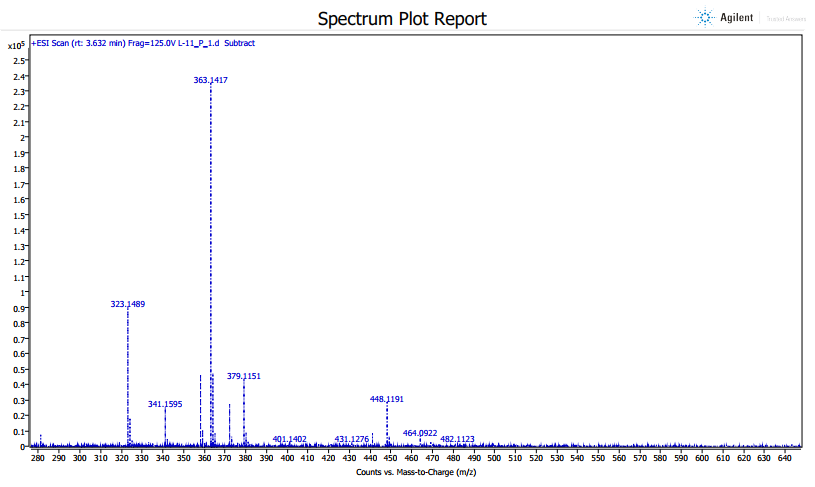


**Figure. S17.**


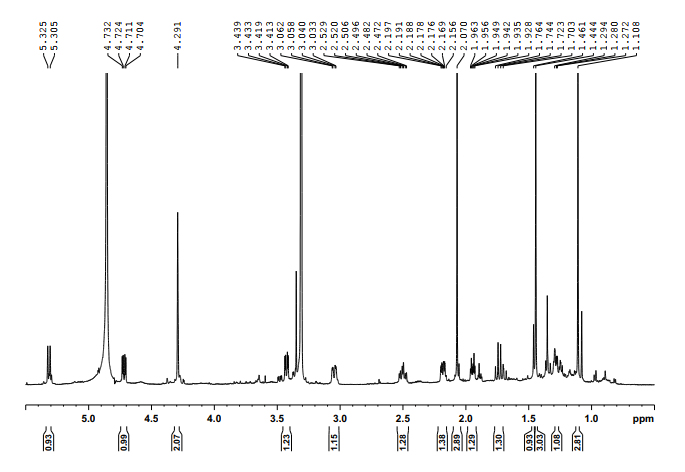


**Figure. S18.**


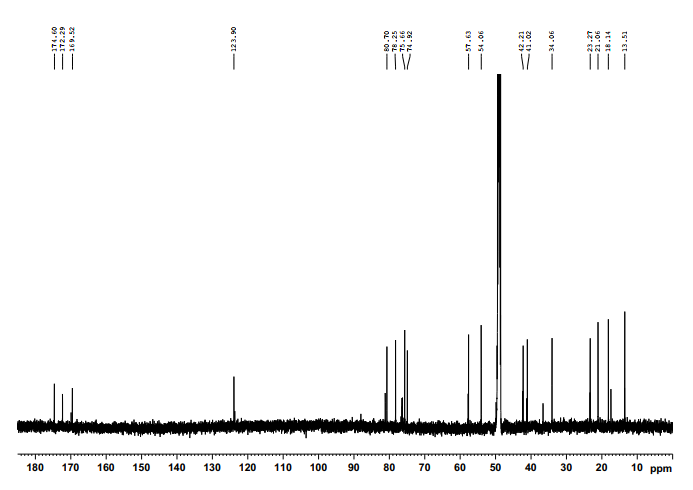


**Figure. S19.**


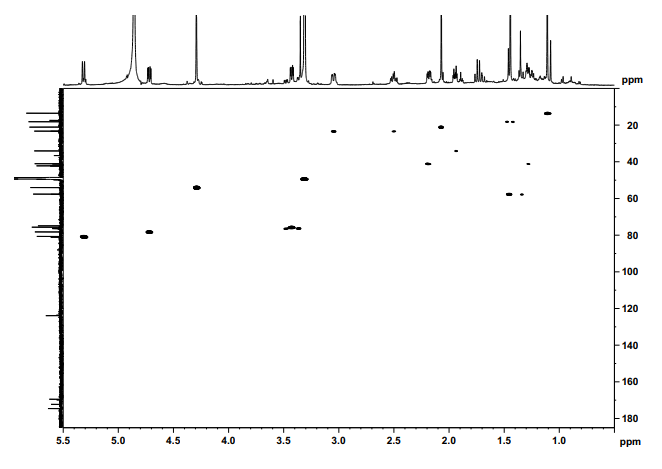


**Figure. S20.**


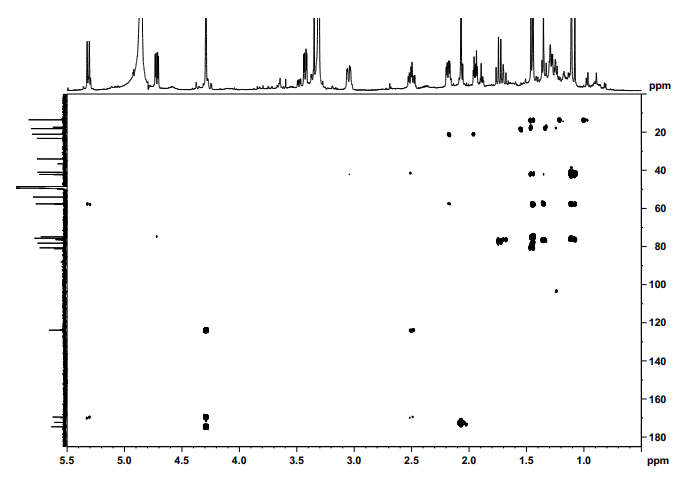


**Figure. S21.**


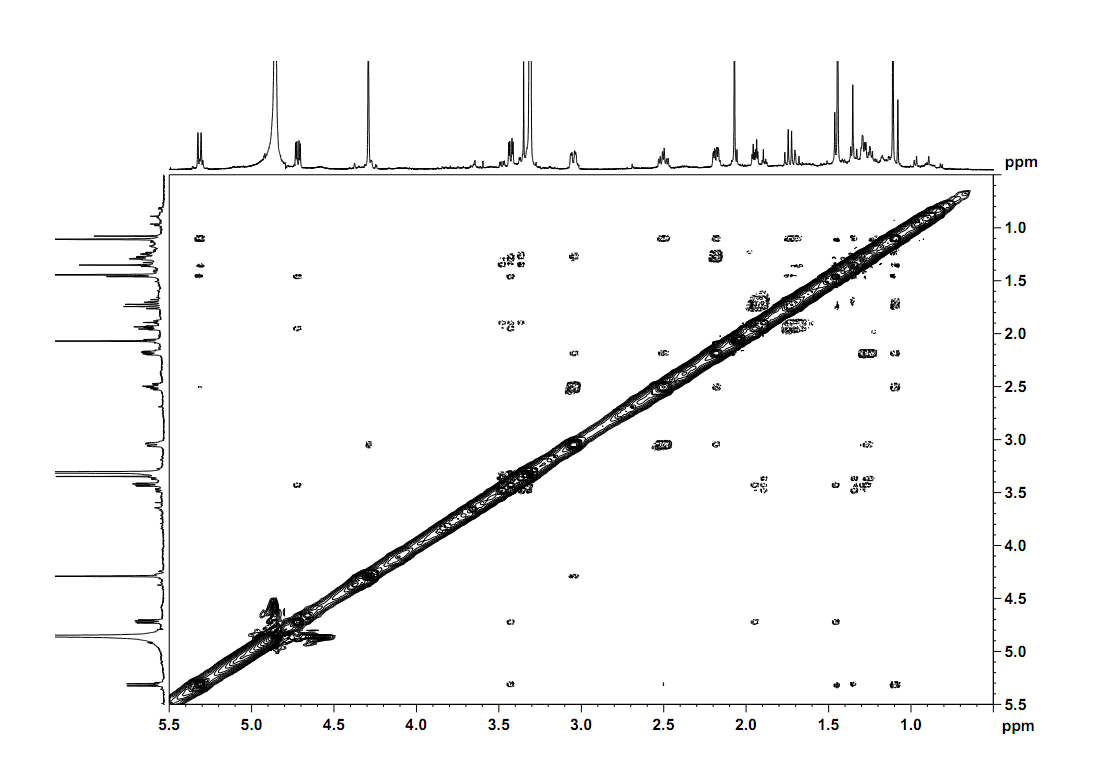


**Figure. S22.**


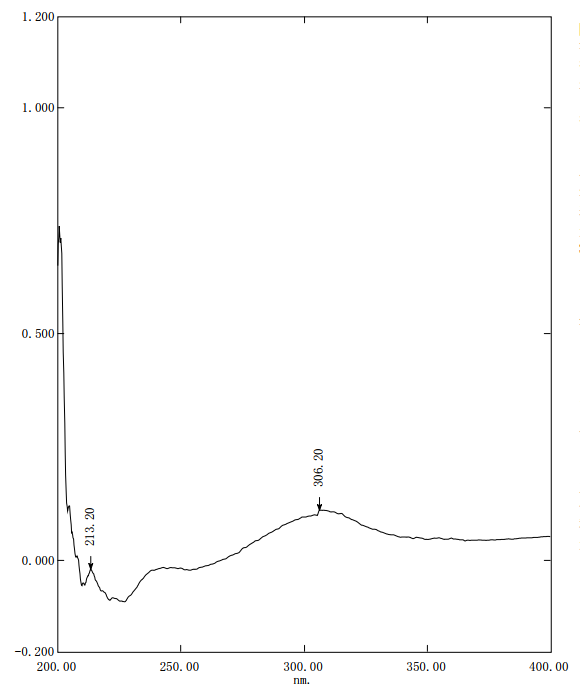


**Figure. S23.**


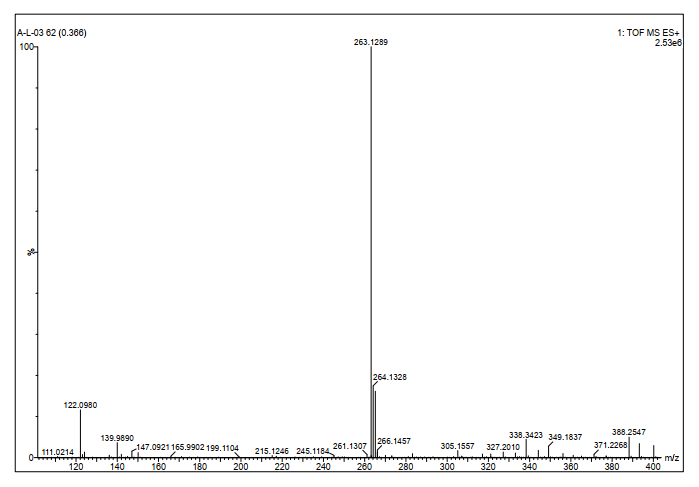


**Figure. S24.**


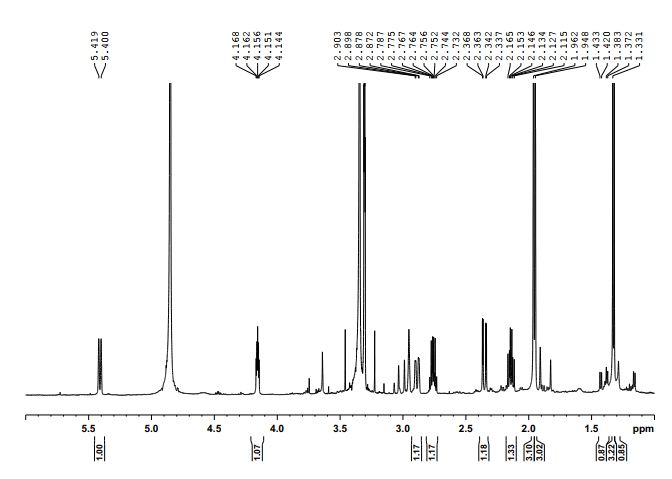


**Figure. S25.**


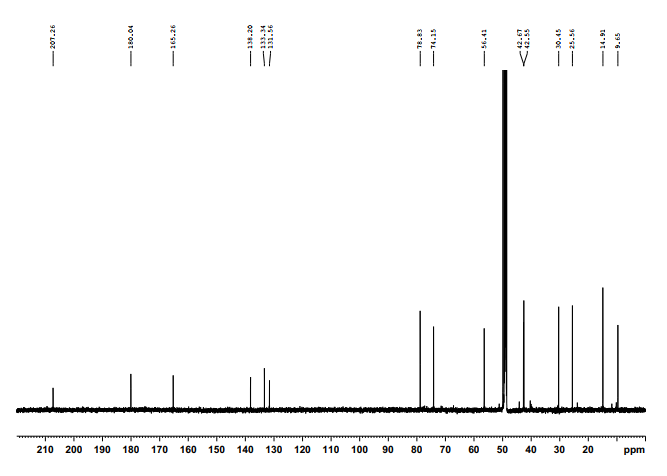


**Figure. S26.**


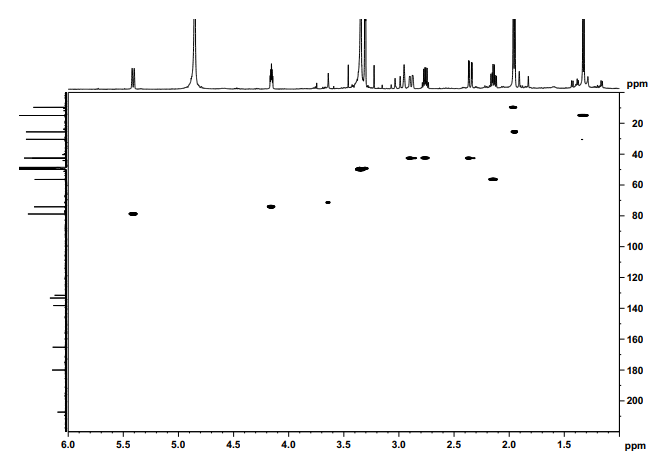


**Figure. S27.**


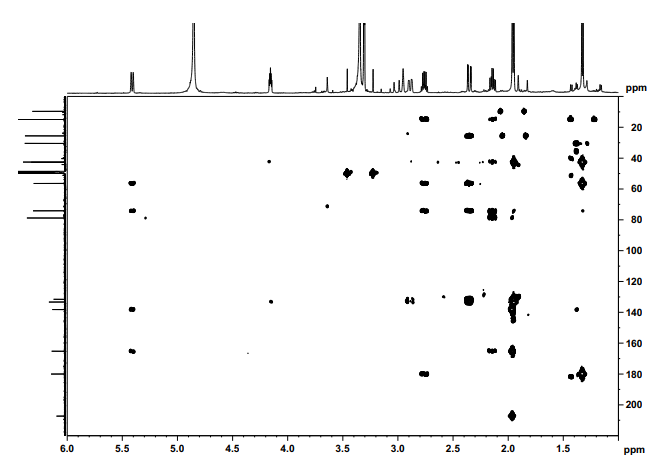


**Figure. S28.**


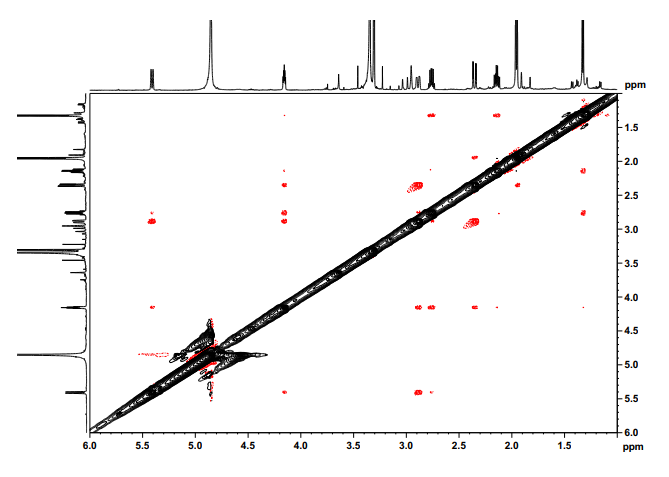


**Figure. S29.**


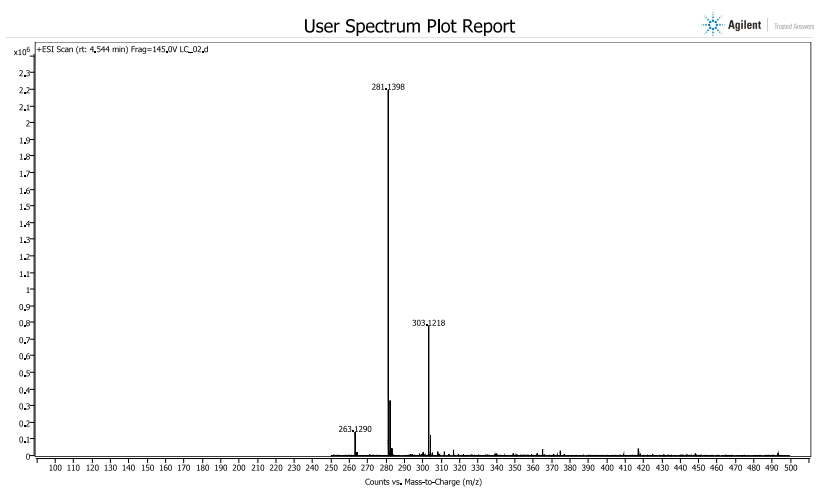


**Figure. S30.**


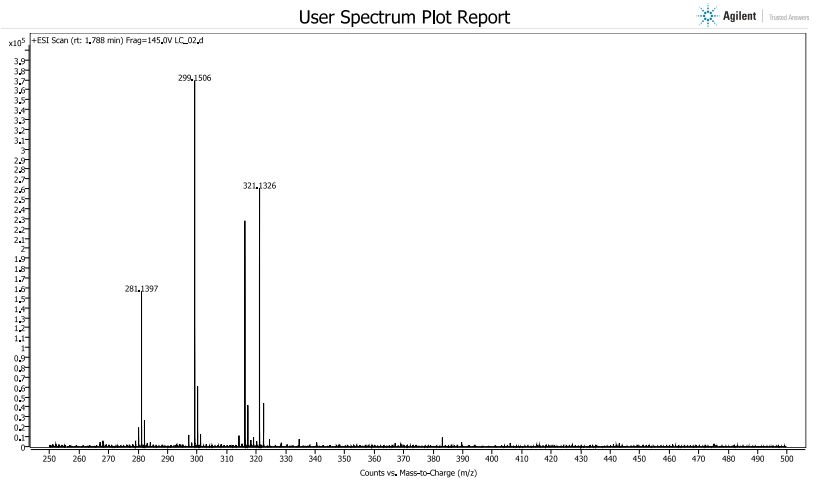


**Figure. S31.**


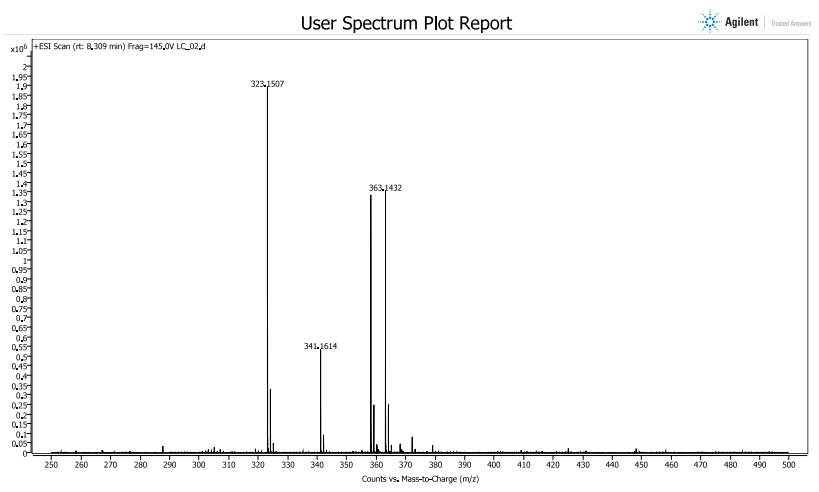


**Figure. S32.**


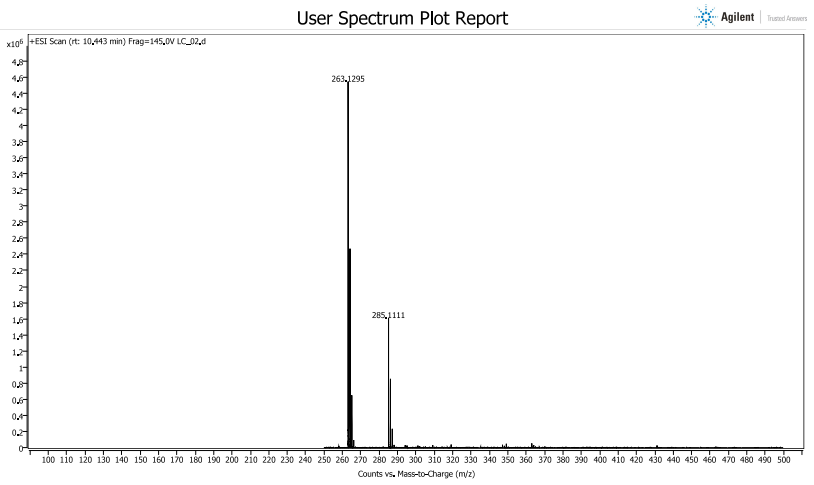


**Figure. S33.**


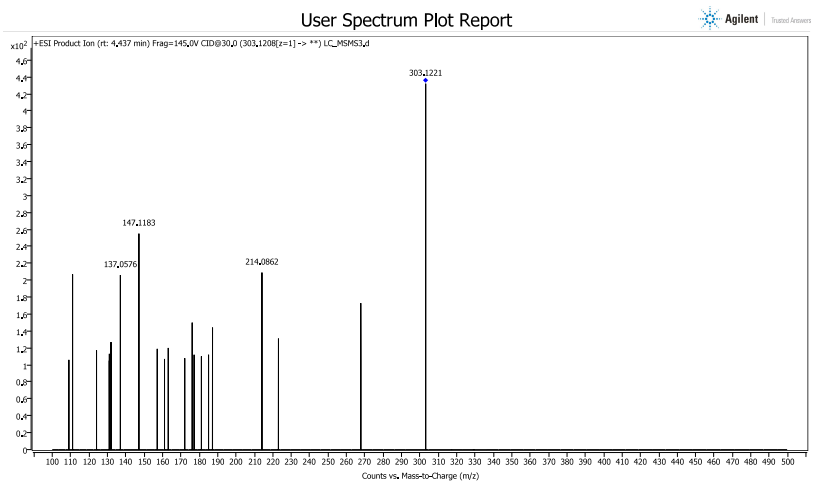


**Figure. S34.**


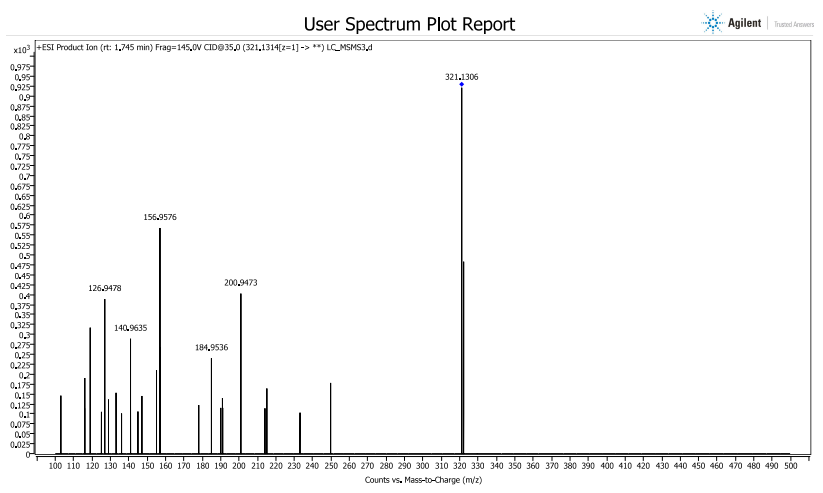


**Figure. S35.**


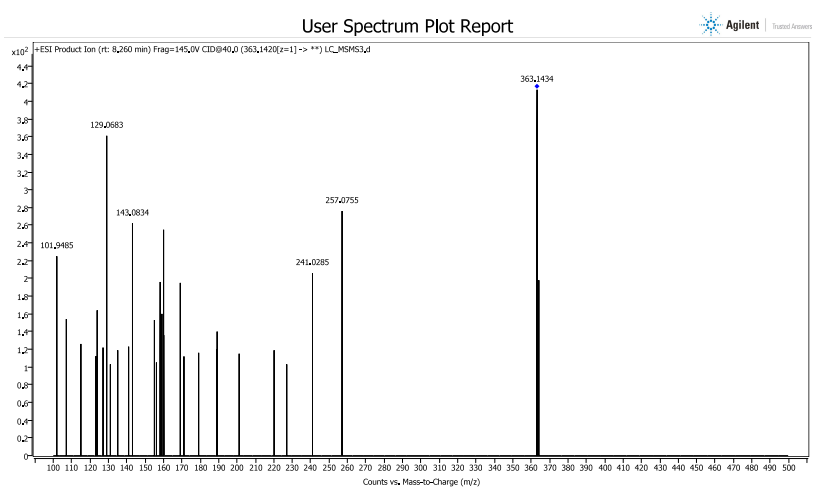


**Figure. S36.**


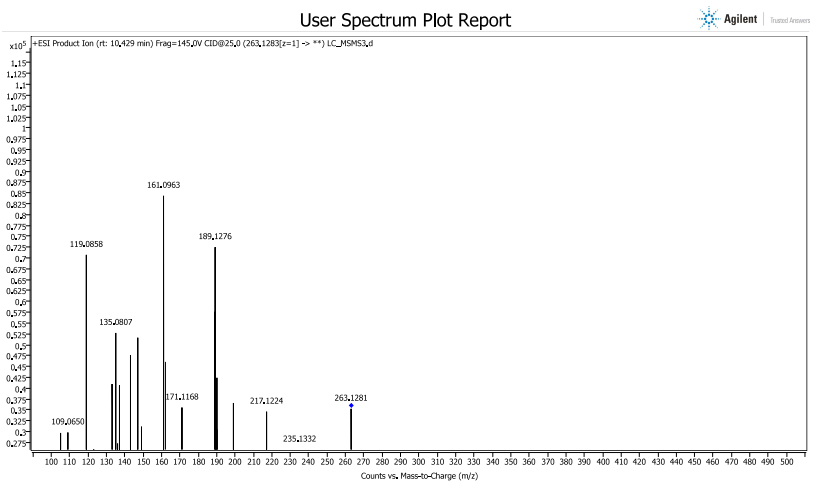

Supplement: Supplementary file 1 [file Data_Sheet_1.docx]
